# Drug-Induced Liver Injury Caused by Kratom Use as an Alternative Pain Treatment Amid an Ongoing Opioid Epidemic

**DOI:** 10.1177/2324709619826167

**Published:** 2019-01-28

**Authors:** Caroline Stokes Osborne, Amanda N. Overstreet, Don C. Rockey, Andrew D. Schreiner

**Affiliations:** 1 Medical University of South Carolina, Charleston, SC, USA

**Keywords:** kratom, DILI, acute liver injury, opioid epidemic

## Abstract

Kratom (*Mitragyna speciosa*) is a prevalent medicinal plant used mainly for the stimulant and analgesic properties provided through multiple alkaloid compounds. Over the past decade, use of kratom has increased despite the limited knowledge of toxicities and adverse side effects. With the current opioid epidemic, both patients and providers are seeking alternative methods to treat both addiction and pain control, and kratom as an alternative means of treatment has increasingly entered the mainstream. In this article, we present the clinical course of a 47-year-old male who developed fatigue, pruritus, and abnormal liver tests (with a mixed hepatocellular/cholestatic pattern) approximately 21 days after beginning kratom. After extensive evaluation including a negligible alcohol history, negative hepatitis serologies, and inconclusive imaging, the patient was diagnosed with drug-induced liver injury (DILI) caused by kratom. Nine months after his liver tests returned to normal, he took kratom again, and after a latency of 2 days, he developed fatigue, pruritus, and loss of appetite along with abnormal liver tests (with the same biochemical profile as previously), consistent with a positive rechallenge. We believe, through the use of the Roussel-Uclaf Causality Assessment Method and expert opinion, that this is a highly likely or definite example of kratom-induced DILI. With the gaining popularity of this drug, it appears that DILI may be an important complication of kratom for providers to recognize.

## Case Description

A 47-year-old man presented to our university-based internal medicine clinic with complaints of dark urine, pruritus, subjective fevers, and fatigue for several days duration. He described subjective fevers with objective measurements ranging from 100°F to 101°F for 2 days with subsequent symptoms of dysuria, urinary frequency, urinary urgency, and darkening of his urine despite large volumes of oral intake. The patient developed generalized malaise, a reduction in appetite, and diffuse pruritus without an associated rash or change in skin color. He reported one episode of nonbloody, nonbilious emesis. He endorsed sick contacts noting his 2 children suffered upper respiratory infection symptoms of cough, rhinorrhea, and sore throat. He denied any recent travel, hospitalizations, or antibiotic use. He took acetaminophen for symptom control but restricted its use to the recommended 3000 mg per day limit. He denied any new or over-the- counter medications including herbal supplements. His previous medical history was notable for obesity (body mass index of 32.68 kg/m^2^), hypertension, prediabetes (previous A1C 6.2%), anxiety, major depressive disorder, and untreated hypertriglyceridemia. His current medications entailed valsartan, metoprolol tartrate, escitalopram, clonazepam, and fexofenadine. His vitals on presentation included a temperature of 36.7°C, heart rate of 53 beats/min, blood pressure of 127/84 mm Hg, and oxygen saturation of 96% on room air. His physical examination revealed nonicteric sclera and sublingual jaundice. He possessed no lymphadenopathy or hepatomegaly. Initial laboratory testing included a point of care urinalysis notable for the presence of urobilinogen and no leukocyte esterase or nitrites. Additional blood work revealed an elevated total bilirubin of 5.8 mg/dL with a direct bilirubin of 4.3 mg/dL, elevated aspartate aminotransferase (AST), alanine aminotransferase (ALT), and alkaline phosphatase (ALP) of 108 U/L, 265 U/L, and 170 U/L, respectively, and an albumin of 3.5 g/dL. His serum creatinine was 1.1 mg/dL with a blood urea nitrogen level of 15 mg/dL. A urinalysis with microscopy noted 30 mg/dL protein, moderate bilirubin, 4.0 mg/dL urobilinogen, and 2 red blood cells per high-power field. The patient was contacted via phone with the laboratory results with an emphasis on the hyperbilirubinemia and elevated aminotransferases. Further history was solicited, and the patient reported a trip to Seattle 3 weeks prior to presentation where he received kratom from a friend. The patient reported that he ingested kratom capsules in an effort to manage his low back pain. Initially, he admitted to only using kratom once. On further questioning, he reported using the substance on multiple occasions, but not daily, at the time of presentation. He again denied alcohol use. His medical stability with normal mentation and robust support system allowed for further evaluation in the outpatient setting.

Further testing revealed a normal prothrombin time and international normalized ratio. Repeat liver tests revealed an uptrend in total bilirubin to 6.1 mg/dL, with a direct bilirubin of 5.1 mg/dL, elevated AST, ALT, and ALP of 114 U/L, 324 U/L, and 148 U/L, respectively, and an albumin 3.5 g/dL. A right upper quadrant ultrasound identified hepatic steatosis without cholelithiasis, cholecystitis, or duct dilation. Additional laboratory tests included an undetectable acetaminophen level, negative Epstein-Barr virus polymerase chain reaction, negative acute hepatitis panel (testing for viral hepatitis A, B, and C), normal α-1 antitrypsin level, and negative antinuclear antibody. He also had a normal thyroid-stimulating hormone of 2.168 U/mL and ceruloplasmin level of 35 mg/dL. The ferritin was elevated at 818 ng/mL with otherwise normal iron studies. Notably, his cytomegalovirus (CMV) IgM antibody index returned positive at 1.7. Laboratory values both 1 week and 2 weeks post index showed improving, but persistent, abnormalities (Table 1). The patient remained out of the hospital during the entire clinical course without complications.

**Table 1.** Serial Serum Liver Tests Results: Initial Presentation and Rechallenge.

Days From First Abnormal	ALT (U/L)	ALP (U/L)	Bilirubin (mg/dL)	AST (U/L)	Event
−21					Agent started
−3					Symptoms started
0	265	170	5.8	108	Sought care, negative HAV, HBV, HCV
2	324	148	6.1	114	Normal PT/INR/PTT; US scan of liver with steatosis
8	149	127	3	36	
16	135	144	1.3	51	
58	60	73	0.6	25	Asymptomatic
					
−3					Agent started
−2					Symptoms started
0	566	211	3.2	185	Sought care, elevated F-actin
3	286	192	4.0	85	Normal PT/INR/PTT
6	238	158	1.4	56	Asymptomatic
19	52	128	0.6	34	
Upper limits of normal	45	150	1.2	34	

Abbreviations: ALT, alanine aminotransferase; ALP, alkaline phosphatase; AST, aspartate aminotransferase; HAV, hepatitis A virus; HBV, hepatitis B virus; HCV, hepatitis C virus; PT, prothrombin time; INR, international normalized ratio; PTT, partial thromboplastin time; US, ultrasound.

Nine months after the resolution of his symptoms and liver test abnormalities, the patient again presented with 2 days of fatigue, loss of appetite, and intense pruritus without rash. A laboratory evaluation revealed a total bilirubin of 3.2 mg/dL, an AST of 185 IU/L, an ALT of 566 IU/L, and an ALP of 211 U/L. After intense questioning, the patient reluctantly admitted to using kratom again, this time in a powder form. This was his first use of kratom since his initial presentation. Given the similar symptoms, biochemical profile, and shortened latency, this constituted a positive rechallenge and further validated the diagnosis of drug-induced liver injury (DILI) caused by kratom. Fortunately, he suffered no impairment of his liver’s synthetic function, and his liver chemistries trended toward normal 3 weeks following rechallenge.

## Discussion

This case provides a clear example of DILI caused by kratom. Initially, this patient’s presentation entailed a clinical scenario of acute liver injury with a number of potentially contributing factors, including nonalcoholic fatty liver disease (NAFLD), CMV hepatitis, and kratom use. However, given the reappearance of symptoms and biochemical abnormalities shortly following a kratom rechallenge, this patient’s diagnosis is kratom-induced DILI (Tables 1 and 2).

**Table 2.** Cases of DILI Caused by Kratom.

Presentation		Our Case		LiverTox #6972^a^	LiverTox #8332^a^	Dorman et al	
		Initial	Rechallenge	Initial	Initial	Initial	Rechallenge
Kratom exposure	Duration of use	Unknown	1 day	24 days	26 days	3 months	1 month
	Latency	18 days	2 days	23 days	25 days	Unknown	2 days
Initial liver tests	Bilirubin (mg/dL)	5.8	3.2	22.4	5.6	9.7	25.6
	AST (U/L)	108	185	—	—	—	—
	ALT (U/L)	265	566	272	126	79	106
	ALP (U/L)	170	211	428	218	270	790
*R* ratio^a^	Initial	5.2 (HC)	8.9 (HC)	2.1 (mixed)	2.1 (mixed)	0.52 (cholestatic)	0.24 (cholestatic)
	Peak	7.3 (HC)	8.9 (HC)	5.0 (mixed)	4.9 (mixed)		

Abbreviations: DILI, drug-induced liver injury; AST, aspartate aminotransferase; ALT, alanine aminotransferase; ALP, alkaline phosphatase; HC, hepatocellular.

a *R* ratio calculated by ([ALT/normal ALT]/[ALP/normal ALP]) using the normal values established at the different laboratories in each of the 4 cases.

Prior to presentation, this gentleman likely had NAFLD given his multiple components of metabolic syndrome (obesity, hypertension, and hyperlipidemia), history of limited alcohol use, and marked steatosis identified on ultrasound. NAFLD is the most prevalent cause of liver disease worldwide, with risk factors including obesity, type 2 diabetes mellitus, hypertension, hyperlipidemia, and metabolic syndrome.^1,2^ NAFLD predisposes patients to greater degrees of injury from other inciting causes, including alcohol, infection, and medication hepatotoxicity.^3,4^ This patient would benefit from lifestyle-directed therapies focused on weight loss, comprehensive management of cardiovascular risk factors, and avoidance of potentially hepatotoxic agents.

Viral hepatitis warrants diagnostic consideration in all cases of acute liver test abnormality but particularly in instances of transaminase elevation. Acute viral hepatitis can result from infection with a number of different pathogens, most notably hepatitis A virus, hepatitis B virus, hepatitis C virus, and typically more indolent, Epstein-Barr virus and CMV. This patient presented with subjective fever, fatigue, and possessed a mildly elevated CMV IgM antibody index during workup. CMV hepatitis is a rare occurrence in immunocompetent patients as it usually causes a self-limiting mononucleosis syndrome and rarely causes organ-specific damage.^5^ CMV hepatitis symptoms predominately involve complaints of right upper quadrant pain and laboratory findings consistent with a hepatocellular pattern of liver injury.^5,6^ Treatment for CMV hepatitis is largely supportive. This patient’s presentation may simply have resulted from CMV infection in the context of NAFLD, but given his immunocompetent status, the absence of lymphadenopathy, the limitations of CMV IgM in acute infection, and the lack of leukocytosis with lymphocytic shift, other diagnoses deserve consideration.^7^

Rapid and comprehensive history taking plays a central role in evaluating abnormal liver tests. Clinicians need to assess patients for critical exposures including alcohol and medication use and pay particular attention to the use of over-the-counter medications and herbal supplements in order to swiftly identify potential cases of DILI. DILI is hepatotoxicity caused by the ingestion of prescription medications, over-the-counter products, and herbal and dietary supplements.^8,9^ Herbal and dietary supplements have especially garnered recent attention given their immense popularity, limited Food and Drug Administration oversight, and linkage to hepatotoxicity. A report from the Drug-Induced Liver Injury Network (DILIN) attributed nearly 15% of DILI cases to herbal and dietary supplements, particularly those used for body building and weight loss.^10^ Diagnosing DILI relies on excluding other potential causes of liver toxicity using clinical, biochemical, and pathologic information obtained via history taking, physical examination, and diagnostic testing.^11^ However, given the subjectivity of this information, achieving an accurate diagnosis can prove difficult. In order to provide objective assessment, clinicians assess the pattern of liver injury in suspected DILI using *R* ratios. Using values obtained at the onset of suspected DILI and calculated by the equation *R* = (ALT/ULN [upper limit of normal]) ÷ (ALP/ULN), *R* ratios help categorize liver injury into hepatocellular (*R* < 2), mixed (2 ≤ *R* ≤ 5), and cholestatic (*R* > 5) patterns. Additionally, clinicians can incorporate this score into the Roussel-Uclaf Causality Assessment Method (RUCAM) instrument, a validated tool for DILI diagnosis.^12,13^ The RUCAM tool applies historical and objective information to provide a clinical likelihood of DILI. However, this tool relies heavily on information regarding the timing between use of the offending agent and the onset of liver injury. In this case, the patient’s history of kratom ingestion evolved over time, highlighting both potential difficulties in obtaining exposure histories and the need to pursue the history meticulously and relentlessly.^14^

Our case shares similar clinical and laboratory features reported in previously reported kratom-induced DILI cases (Table 2).^15-18^ The chief complaints of fatigue, nausea, pruritus, and dark urine in our patient with a latency of 21 days after the ingestion of kratom resembles previous cases.^16-18^ Objectively, our patient first presented with an initial *R* ratio 5.2 suggestive of a hepatocellular pattern of injury with marked hyperbilirubinemia (5.8 mg/dL, 4.8 times the upper limit of normal). The *R* ratio peaked at 7.3 and the total bilirubin at 6.1 mg/dL. Using the RUCAM instrument, patient’s data in the initial presentation resulted in a score of +6, suggesting a “probable” diagnosis of DILI.^13^ This cumulative score included points for time to onset (5-90 days, +2), course (ALT decreasing >50% within 30 days, +2), exclusion of other causes of liver injury (all save CMV, +1), and previous information on hepatotoxicity (LiverTox reports, +1). When the patient returned with symptoms and an *R* ratio of 9 after another instance of kratom use, the likelihood of DILI significantly heightened. Using the RUCAM again, the positive rechallenge with a short latency and doubling of ALT (an more in this case) in response to the agent alone adds a +3 to the score, moving the total to a +9, which equates to a “high probability” or “definite” case of DILI caused by kratom. Between our case and previous reports, kratom appears to be able to cause any biochemical injury pattern ranging from cholestatic to hepatocellular (Table 2).

Multiple clinically significant confounders apply to our case, as are often found in most DILI cases.^19^ As previously noted, the patient likely had NAFLD at baseline, which could potentiate liver injury of any variety. Testing also revealed a positive CMV IgM, providing a diagnostic alternative despite the test’s limitations in specificity and the incomplete clinical picture. Additionally, there may be contributions from other pharmacologic exposures in this case. Our patient admitted to taking acetaminophen at the start of his symptoms but had undetectable levels found on the day of presentation. The patient also took a long-term selective serotonin reuptake inhibitor. Given the serotonergic-related activity, the capacity to inhibit cytochrome P450 enzymes, and the liver metabolism of both agents, the potential for drug-herb interactions in this case loom large.^20^

The United States is in the midst of an opioid epidemic. As the government, medical societies, and individual practitioners address this public health concern, other legal means of analgesia are increasing in popularity.^21-23^ Hundreds of online retailers throughout the United States and Europe advertise and sell countless herbal products for stimulant and analgesic purposes. One of these herbal products frequently advertised as an opium substitute is kratom (*Mitragyna speciosa*).^24^ *Mitragyna speciosa* is a native plant to Southeast Asia and originally used by manual laborers as a means to combat fatigue and enhance work productivity due to its stimulant and analgesic properties.^25^ The opioid-like properties stem from multiple alkaloid compounds, namely, mitragynine and 7-hydroxymitragyine, which bind to the *mu, kappa, and delta* receptors.^25^ The pharmacology of kratom is complex as it also interacts within the serotonergic and adrenergic pathways.^25^ Kratom provides a stimulant effect at low doses (1-5 g) and opioid-like effects at high doses (5-15 g). Kratom leaves are consumed through several means: brewing as a tea, smoked, chewed, or processed into a form of an ingestible capsule.^25,26^ The mechanism of ingestion plays a critical role in evaluating the potential harm of this compound, as a recent study found significantly higher concentrations of active metabolites in commercially available kratom supplements compared with raw, unprocessed *Mitragyna* leaves.^27^ The use of concomitant medications is also of concern, as *Mitragyna* may inhibit cytochrome P450 enzymes and potentiate harmful herb-drug interactions (ie, serotonin syndrome).^20^ Symptoms observed during withdrawal from kratom resemble those of opioid withdrawal with rhinorrhea, myalgias, dysphoria, decrease in appetite, and diarrhea.^21,25^ Nonetheless, due to its analgesic properties, companies advertise and patients consume kratom either as a “natural” means of chronic pain control or opioid withdrawal symptom management.^21,25^ Many uncertainties surround kratom with regard to its safety profile, side effects, potential drug interactions, and overdose threshold.^21-23^ A Centers for Disease Control and Prevention report from July 2016 noted a 10-fold increase in reports to poison centers around the United States regarding the ingestion of kratom.^28^ Despite the growing popularity and concomitant safety concern of kratom, literature regarding the safety profile and side effects of kratom remains limited.

## Conclusion

As the opioid epidemic in the United States continues, patients may turn to unconventional means of pain control. Herbal supplements, such as kratom, are gaining in popularity in part due to ease of access but also due to the presentation of kratom as a safe, natural way to self-taper and control withdrawal symptoms. Patients may be turning to kratom to manage symptoms of withdrawal when they lack access to medication-assisted treatment for opioid use disorder. Unfortunately, the safety of kratom is not confirmed, and the literature regarding its safety profile and side effects is limited. As patients and providers work together in a patient-centered paradigm to curb opioid use and find alternative adjuvants for pain control, herbal supplements with opioid properties like kratom will have to be considered. Providers must diligently inquire about herbals and supplements now when taking a pain history. Additionally, providers must seek to educate patients regarding the lack of kratom’s safety profile, lack of known overdose threshold, and lack oversite over the manufacturing of kratom in order to continue an open dialogue between patient and provider.
